# *Pseudosigmoidea ibarakiensis* sp. nov., a Dark Septate Endophytic Fungus from a Cedar Forest in Ibaraki, Japan

**DOI:** 10.1264/jsme2.ME13002

**Published:** 2013-09-04

**Authors:** Ousmane Diene, Wei Wang, Kazuhiko Narisawa

**Affiliations:** 1 Direction de la Protection des Végétaux, Km 15 Route de Rufisque, BP: 20054, Dakar, Sénégal; 2 Department of Bioresource Science, College of Agriculture, Ibaraki University, 3–21–1 Chuo, Ami, Ibaraki, 300–0393, Japan

**Keywords:** *Pseudosigmoidea*, Hyphomycetes, taxonomy, endophyte, environmental stress

## Abstract

A dark septate fungus of *Pseudosigmoidea*, Hyphomycetes, was recovered from forest soil in Ibaraki prefecture, Japan. The isolate is characterized by pale to brown conidia with up to 8 septa measuring 68–132 × 4–7.9 μm. It is also unique in producing conidia borne by long conidogenious cells in agar medium with or without water, compared to *P. cranei*, which must be immersed in water to sporulate. Morphological analysis indicated that the isolate is distinct from *P. cranei* and is described as a new species, *P. ibarakiensis* sp. nov. Pathogenicity tests of Chinese cabbage and cucumber seedlings indicated that the fungus grows as an endophyte and colonizes, inter and intracellularly, the root epidermal and cortical layers without causing apparent disease symptoms in the host. This endophyte showed the ability to support cucumber plant growth under conditions where NaNO_3_ was replaced by organic nitrogen but also conferred to Chinese cabbage the ability to grow at low pH. It also became successfully established in six other plants, including the Brassicae, Solanaceae, Poaceae, and Liliacea families, suggesting its adaptability to a broad range of host plants.

Fungal endophytes are fungi that colonize the internal tissues of living plants without causing any external disease symptoms. Such an association, which can go back millions of years (20, 30), benefits both the fungus and host plant. Of these fungi, dark septate endophytes (DSEs), characterized by dark-pigmented and septate mycelia, particularly confer traits that improve their hosts’ tolerance to unfavorable environmental conditions (14, 17).

When isolated in pure cultures, colonies of DSEs are generally nondescript, range in color from olivaceous to brown or almost black, and often lack conidia or other taxonomically distinctive characteristics (17). DSE fungi consist of a diverse group, are distributed worldwide, and are associated with a broad range of hosts that include around 600 species of plants from 320 genera and 114 families, such as Poaceae and Solanaceae, especially under harsh climatic conditions (1, 17, 33). To date, however, only 9 DSE species, *Chloridium paucisporum*, *Leptodontidium orchidichola*, *Phialocephala dimorphosphora*, *P. fortinii*, *Phialophora finlandica*, *Meliniomyces variabilis*, *Heteroconium chaetospira*, *Veronaeopsis simplex*, and *Helminthosporium velutinum*, have been reported (6, 17, 22, 23). Most of these DSE species were isolated from northern coniferous and boreal forests in nutrient-poor soils, except *Heteroconium chaetospira*, *Veronaeopsis simplex*, and *Helminthosporium velutinum*, three species of different genera, all isolated from coniferous rainforests, including freshwater in Japan. Furthermore, recent studies showed multiple useful traits, such as nitrogen provision, growth promotion, and disease suppression, in host plants to be associated with these three taxa (6, 18, 19, 35). Based on these findings, DSE populations in Japan may be more diverse than previously thought and include other undescribed species. However, more new isolates are needed to support this assumption.

Recently, the ability of some freshwater hyphomycetes to grow as endophytes has been documented (32). The freshwater hyphomycete genus *Sigmoidea* has up to four species, including *S. prolifera*, *S. auratiaca*, *S. praelonga*, and *S. contorta* (15). These fungi are known from their DSE features, such as the color of hyphae, behavior in the hosts, etc. *S prolifera* has had a very evolving taxonomy at the genus level. Originally described as *Flagellospora* Ingold when *F. prolifera* Pertesen was first described (27), it was reconsidered by Crane (4) as *Sigmoidea*, to accommodate *S. prolifera* Crane. Recently, Ando and Nakamura (2) established from *Sigmoidea* the genus *Pseudosigmoidea* Ando and Nakamura to accommodate *P. cranei*, formerly known as *S. prolifera*. Molecular studies by Jones *et al.* (15) confirmed that the two specimens represented two different taxa. Although the new taxonomic position of this *Pseudosigmoidea* is correct, only a single species has been described based on existing specimens and very little is known about its ecological role and distribution.

DSE behavior is based upon the intra and/or extracellular colonization of host plants tissues with no apparent negative effects (18). However, depending on the fungal species, the colonized host tissues can differ. *Heteroconium chaetospira*, for example, when inoculated into Chinese cabbage, colonized both epidermal and cortical layers but did not reach the vascular cylinder (21) whereas *Phialocephala fortinii* can occasionally enter the vascular cylinder of host root tissues (40). The outcome of these interactions is beneficial to host plants and can include the provision of nutrients through the transfer of nitrogen and uptake of phosphorus (9, 35, 36).

Consequently, a new search was initiated to look for endophytic fungi from cedar forest soil using tomato and Chinese cabbage as bait plants. As a result, we obtained 2 isolates of *Meliniomyces* and *Pseudosigmoidea* showing DSE features.

Morphological examinations supported by molecular data revealed that the isolate of *Pseudosigmoidea* did not fit the single existing species *P. cranei* of this genus. In the present paper, we describe a new DSE species, *Pseusigmoidea ibarakiensis* Diene & Narisawa. In addition, the endophytic nature of the fungus was studied to test the ability of its host plants to use organic nitrogen and to tolerate low pH.

## Materials and Methods

### Sample collection and fungal isolation

Twelve soil samples (three per site) were collected from four different natural forests surrounding the town of Ami in Ibaraki prefecture, Japan. All the sites were characterized by the presence of cedar trees. Sampling was performed according to Narisawa *et al.* (22) at a depth of 5 to 20 cm and samples were kept in polyethylene bags and stored at 4°C for a maximum of 10 d prior to utilization. Soil samples from each site were separately mixed with autoclaved potting soil (Peat pot) at a ratio of 1/3 (v/v) to serve as composite soil for baiting fungal endophytes, using tomato c.v. Hausu Momotaro and Chinese cabbage c.v. Musou (Takii Seed, Kyoto, Japan), two easily grown annual plant species.

For each bait plant, five-day-old seedlings grown aseptically on water agar were transplanted into 10.5-cm diameter pots containing approximately 400 mL composite soil. Each collection site was considered as a bloc containing three replicate pots for each soil sample.

Three weeks after transplantation, the roots collected from young tomato and Chinese cabbage plants in each replicate were washed with running tap water to remove debris and cut into approximately 1-cm segments. Fifteen randomly chosen fragments of each bait plant were washed three times in a 0.005% solution of Tween 20 (Wako Pure Chemical, Osaka, Japan), followed by three rinses in sterilized distilled MilliQ water (Avarium/Sartorius Stedim Biotech, Gottingen, Germany). Segments air-dried overnight were plated in 9-cm plastic Petri dishes (three segments in each dish, five dishes per replicate) with 50% corn meal agar medium (cornmeal infusion, 1 g; Bacto agar, 7.5 g L^−1^ [Difco/Becton Dickinson, MD, USA]). During incubation at 23°C, for identification purposes, single fungal colonies were isolated and grown on cornmeal malt yeast extract medium (cornmeal infusion, 8.5 g; Bacto agar, 7.5 g; malt extract, 10 g; yeast extract [Difco/Becton Dickinson], 2 g L^−1^) in 6-cm Petri dishes.

### Morphological observation and identification

To provide good observation conditions, slide cultures were made. Small pieces, approximately 3×3 mm, of Publum agar (Mead Johnson mixed Publum [Canadian Post Corporation, Ontario, Canada], 25 g; Bacto agar, 5 g; MilliQ water, 250 mL) were sandwiched between two 18×18-mm cover glasses (Matsunami Glass, Osaka, Japan) and placed in a 9-cm water agar plate to provide humidity. After 2 to 4 weeks, when the culture had grown sufficiently, the publum agar was carefully removed and cover glasses were appropriately mounted on 76×26 mm micro slide glasses using PVLG (polyvinyl alcohol, 16.6 g; lactic acid, 100 mL; glycerin, 10 mL [Wako Pure Chemical]; MilliQ water, 100 mL) mounting medium. Conidiogenous cells and conidia were measured under a light microscope (BX51; Olympus, Tokyo, Japan) with UPlanFLN FLN100x/1.30 oil.

### DNA extraction, amplification, sequencing and analysis

The nuclear small-subunit rRNA gene was targeted for clear molecular identification of the fungal isolate focusing on the 18S rRNA gene region. The genomic DNA was extracted from mycelium grown on oatmeal medium using the Prepman Ultra Sample Preparation Reagent Protocol (Applied Biosystems/Life Technologies, Carlsbad, CA, USA). Primers NS1 and NS4 (35) were used to amplify the 18S rRNA gene region by polymerase chain reaction (PCR). The reaction mixture (50 μL) contained 5 μL of 10x *rTaq* DNA polymerase buffer, 2.5 μL of each primer, 4 μL dNTP mixture, 35.375 μL MilliQ water, 0.125 μL *rTaq* DNA polymerase, and 0.5 μL template DNA. Amplification was carried out using a Takara PCR Thermal Cycler Dice (model TP 600; Takara Bio, Shiga, Japan) as follows: 94°C for 4 min, 35 cycles of 94°C for 35 s, 52°C for 55 s and 72°C for 2 min, followed by a final 10 min extension at 72°C. For sequencing, the 10 μL reaction mixture (Big Dye Terminator ver. 3) contained 1.5 μL of 5×sequencing buffer; 1 μL primer (3.2 μM), 1 μL pre-mixture (kit), 6.2 μL sterilized MilliQ water and 0.310 μL template purified DNA. The sequencing conditions were: 96°C for 2 min, 25 cycles of 96°C for 10 s, 50°C for 5 s, and 60°C for 4 min.

### Pathogenicity screening

In order to distinguish non-pathogenic and endophytic fungi from pathogenic and other saprotrophic fungi, isolates were screened by means of a pathogenicity test. Fifteen isolates showing diverse morphology were selected as representative of each group of fungi, with priority given to slow growing isolates that started developing after at least 7 to 10 days of incubation. Pathogenicity tests were carried out after growing the fungi in 6-cm Petri dishes filled with oatmeal medium (oatmeal, 10 g L^−1^; Bacto agar, 1 g L^−1^) enriched with nutrients (MgSO_4_·7H_2_O, 1 g L^−1^; KH_2_PO_4_, 1.5 g L^−1^; NaNO_3_, 1 g L^−1^ [all Wako Pure Chemical]). The method used has been modified from Narisawa *et al.* (24). To test nitrogen sources, NaNO_3_ (Wako Pure Chemical), at 1 g L^−1^ was replaced by the selected nitrogen source as described below at the desired concentration, but other nutrients remained unchanged. After the plates were covered by sufficient colonies, surface-sterilized 2-d-old seedlings of Chinese cabbage were transplanted (three per plate) onto the growing colony and the whole set placed into a sterile plastic pot and incubated for two to three weeks at 23°C with a 18 h:6 h (L:D) photoperiod at 600 lux. Symptoms were evaluated according to an index varying from 0 to 3 (0: no visible symptoms; 1: slight yellowing; 2: yellowing and late growth; 3: wilting or death), the plants were harvested and oven-dried at 40°C for 48 h, and their dry weights were measured and compared to control (non-inoculated) plants. Only plants with no visible symptoms (index 0) with a dry weight greater than or equal to control plants were selected as potential endophytic candidates to undergo further confirmation tests.

### Inoculation, fungal re-isolation and colonization observation

After successful pathogenicity testing, the need arises to prove the colonization of fungal isolates on host plants. It should be possible to re-isolate fungal isolates from colonized but obviously not colonized segments. Inoculation and re-isolation tests were carried out on cucumber c.v. Jibai (Takii Seed). However, to verify the host specificity of the representative DSE isolate, I.4-2-1, re-isolation tests were extended to six other plant species: canola, sweet sorghum c.v. “FS902”, tomato, asparagus, beans, and onion c.v. Soniku of the Brassicacae, Poaceae, Solanacae, Asparagaceae, Fabaceae, and Amaryllidaceae, respectively. Inoculation was performed as described before. Surface sterilization was performed as described for pathogenicity testing. To determine the endophytic nature of fungal isolates, infected hyphae of the inoculated fungi in 3-week-old cucumber seedling roots were observed after washing, cross sectioned, and stained in 50% acetic acid solution containing 0.005% cotton blue under a light microscope.

### Nitrogen source test

In order to identify the effect of nitrogen on fungal infection, a nitrogen source test was conducted. The fungus was grown for 2 weeks in 6-cm Petri dishes filled with oatmeal medium as described before but NaNO_3_, 1 g L^−1^ was replaced by one of the selected nitrogen sources of amino acids, such as l-valine, l(−)-phenylalanine, and l-leucine (Wako Pure Chemical), at a concentration of 20 mg L^−1^. Disinfected cucumber seeds were directly sown on the respective plates. All other procedures were the same as those in the pathogenicity tests. Amino acid concentrations were optimized using l-valine at concentrations of 5, 10, 15, 20, 25, 30, and 40 mg L^−1^.

### pH tolerance tests

A pH tolerance test was undertaken to investigate the effect of pH on fungal infection and plant growth. Inoculation of the fungi, pathogenicity ratings and evaluation of plant growth were performed as described previously using Chinese cabbage under various pH conditions. pH was set to a range varying from 3 to 8 using a Docu-pH^+^ meter (Sartorius Mechatronics, Goettingen, Germany) and adjusted with KOH and HCl (both Wako Pure Chemical). These pH-adjusted plates were inoculated with the fungus and incubated at 23°C for two weeks. Subsequently, Chinese cabbage seedlings were transplanted to the same plates to evaluate their host biomass. All other procedures were similar to those used in the pathogenicity tests.

### Data analysis

The mean dry biomass of each treatment was calculated and analyzed with one-way ANOVA. Differences among treatment means were detected with Tukey’s honestly significant difference test.

## Results

### Sample collection and fungal isolation

A total of 210 tomato root segments were analyzed, yielding 93 slow growing isolates, representing a rate of fungal isolation of 44% (Table 1).

### Pathogenicity screening

**Fig. 1:**
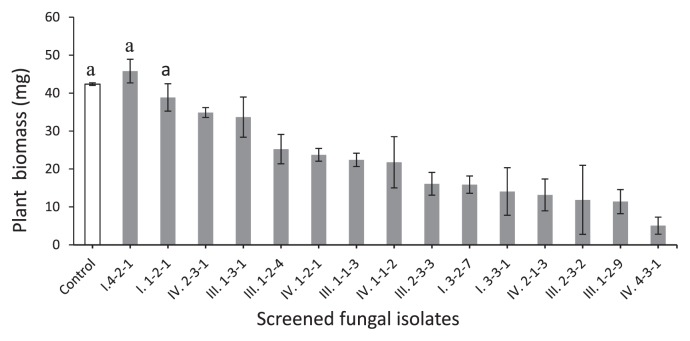
Dry weight of Chinese cabbage seedlings grown on oatmeal agar enriched with MgSO_4_·7H_2_O, KH_2_PO_4_, and NaNO_3_, and inoculated with fifteen selected isolates (Control: without fungal isolate). Data are the mean±SD, n=3. Columns with the same letters are not significantly different (P<0.05) following Tukey’s honestly significant difference test.

Fifteen isolates showing diverse morphology and selected as representatives of each morphological group were used for endophyte screening. Four of the isolates were from Site I, six from Site III and five from Site IV. None of the isolates from Site II was tested because they all matched the morphological characteristics of those from the other three sites. In inoculation test results, only two of the fifteen isolates tested (approximately 13%) were not pathogenic to Chinese cabbage seedlings (Fig. 1). These two isolates, I.1-2-1 and I.4-2-1, caused no sign of disease or decay in Chinese cabbage seedlings and were assigned a pathogenicity rate of 0. The weight of dried plants was 38.8±3.6 mg and 45.8±3.1 mg for I.1-2-1 and I.4-2-1, respectively, and showed no significant difference from the control plants, 42.3±0.3 mg (Fig. 1). Most of the isolates tested (over 86%) were pathogenic to Chinese cabbage at various pathogenicity rates, ranging from 1 to 3.

Isolate I.1-2-1 was concluded as *Meliniomyces variabilis* based on 99% similarity of 18S rRNA gene sequence with the endophytic isolate (access number AY838792) from Hambleton and Sigler (12), representing its first report in Japan, although it is believed to have a circumboreal distribution. The remaining isolate, I.4-2-1, did not match the characteristics of any described species.

### Morphological characteristics

**Fig. 2:**
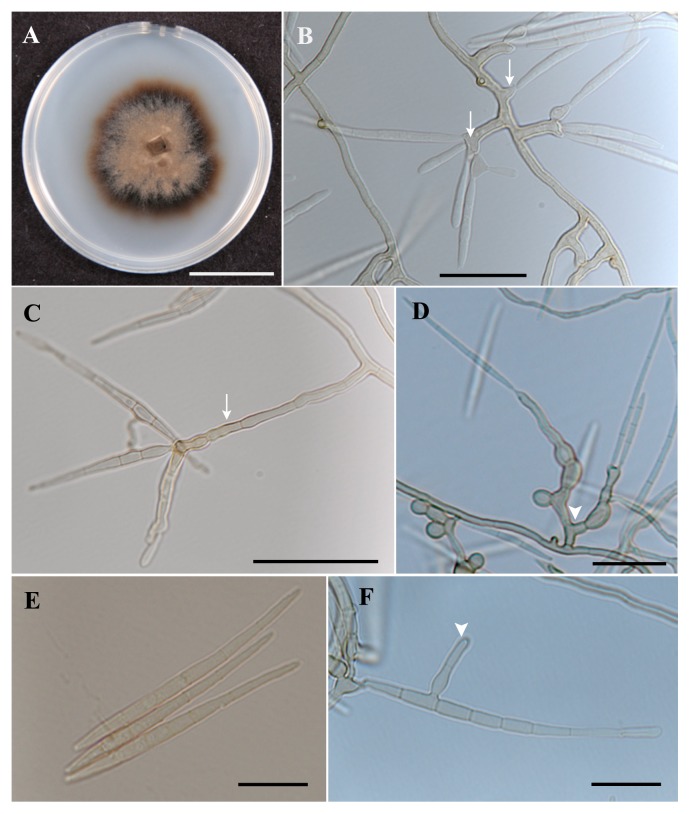
A–H, Colonial and microscopic morphology of *Pseudosigmoidea ibarakiensis* sp. nov. (A) Colonial growth on oatmeal [OMA] after three weeks at 23°C. (B–D) Light micrographs showing conidiogenous cells rising as lateral branches from mycelium in Publum agar medium [arrows indicate simple conidiogenous cells of different sizes; arrowheads indicate branched conidiogenous cells]. (E–F) Conidial shape: group of simple conidia (E) and secondary conidial formation (F). Bars A. 20 mm; B. 20 μm; C. 30 μm; D. 20 μm; E. 20 μm; F. 20 μm.

Colonies of I.4-2-1 were dark brown when grown on OMA, 50% CMMY, Malt Agar and LCA media. The isolate turned the CMMY medium slightly darker. This fungus is characterized by an irregular, flat, smooth, and with an entire margin colonial morphology. Growth was relatively slow in all media and reached around 20 mm after 4 weeks at 23°C (Fig. 2A). This fungus grows best between 20 to 25°C.

Mycelial hyphae were hyaline to brown, septate, branched, and 1.7–3.3 (2.33) μm in diameter. Conidiogenous cells developed as lateral branches (Figs. 2B and 2C) ranging in size from short to long; 7–60 × 2.5–5.5 μm (Fig. 2C), and were polyphialidic, producing up to five conidia (Figs. 2B, 2C, and 2D). The conidial ontogeny was enteroblastic. These conidiogenous cells were mostly simple (Fig. 2B) but could also be branched (Fig. 2D). Conidial formation occurred giving elongate to slightly curved, multiseptate conidia ranging from 68 to 133 μm in length and 4 to 8 μm in width (Fig. 2E). Secondary conidial formation was also observed on the tip of the primary conidia or laterally (Fig. 2F). These morphological characteristics match those of the genus *Pseudosigmoidea*, characterized by enteroblastic and phialidic conidiogenesis. The morphological differences from *P. cranei* are related to the sizes of conidiogenous cells and conidia. Conidiogenous cells are longer in I.4.2.1 with a range of 7–60 × 2.5–5.5 μm, whereas in *P. cranei*, they were 1–13 × 2–3 μm. Moreover, the conidia varied in size from 68–133 × 4.03–7.94 μm for isolate I.4-2-1 compared to 29–116.5 × 1.5–2.5 μm for *P. cranei*. Lastly, *P. cranei* requires immersion in water to sporulate, in contrast to isolate I.4-2-1, which can sporulate without immersion.

The isolate has been deposited in the NITE Biological Resource Center of Japan under code NBRC 107891.

The sequence of 18S rRNA gene of isolate I.4-2-1 showed high similarity (99 to 100%) to *Troposporella fumosa* (access number AY856953), *Helicoma monilipes* (access number AY856920), and *H. olivaceum* (access number AY856925). However, the fairly low alignment scores of these database sequences, covering only 75 to 77% of the query sequence, indicated weak relatedness of this isolate to the above species. In addition to these results, the morphological analyses showed that this isolate is quite different from the two genera *Troposporella* and *Helicoma*.

For *P. cranei* (access number DQ104808) on the other hand, a sequence similarity of 97% supported by a high alignment score (100%) of the query sequence was observed. We concluded that this isolate belongs to a different species from *P. cranei*, because the similarity between them is quite low.

The sequence was deposited in the DNA Database of Japan (DDBJ) accession number AB697751. To accommodate this isolate, the following species is proposed: *Pseudosigmoidea ibarakiensis* O. Diene, & K. Narisawa, sp. nov.

#### Taxonomy

**Pseudosigmoidea ibarakiensis** O. Diene, & K. Narisawa, sp. nov. Fig. 2A–F

Mycobank MB804932

*Mycelial hyphae* hyaline to brown, septate, branched, 1.7–3.3 (avg. 2.3) μm in width. *Conidiogenous cells* simple to branched, develop as lateral branches 7–60 × 2.5–5.5 μm, polyblastic, producing up to five conidia. They develop either directly or borne by conidiophores that can reach over 60 μm (Fig. 2C). The conidial ontogeny was enteroblastic.

*Conidia* elongated, slightly curved, up to 8 septate, 68–133 × 4–8 μm (Fig. 2E). Secondary conidia terminal to lateral, occurring from primary conidia (Fig. 2F). Some chlamydospores in a chain are produced whose diameter ranged 4–8 (5) μm.

*Etymology. ibarakiensis* in reference to the Japanese prefecture where the fungus was isolated.

*Holotype*. NIAES H-20615, a dried culture deposited in the herbarium of National Institute for Agro-Environmental Sciences (Ibaraki, Japan).

*Type*. Isolate I.4-2-1 (NBRC 107891) obtained from natural forest soil in Ibaraki, Japan in 2008.

*Distribution.* only at the type location in Ibaraki.

### Inoculation, fungal re-isolation and anatomic observations

After 3 weeks of incubation, I.4-2-1 was successfully established in cucumber and Chinese cabbage seedlings and could be recovered from infected roots. It was also successfully established with no disruption of growth in canola, sweet sorghum, tomato, asparagus, beans, and onion. There was some variation in the re-isolation rate depending on the plants but broadly the rate was high in all 6 plant species tested and ranged between 86 and 100%. The maximum rate, 100%, was observed for sorghum, tomato and cucumber.

**Fig. 3:**
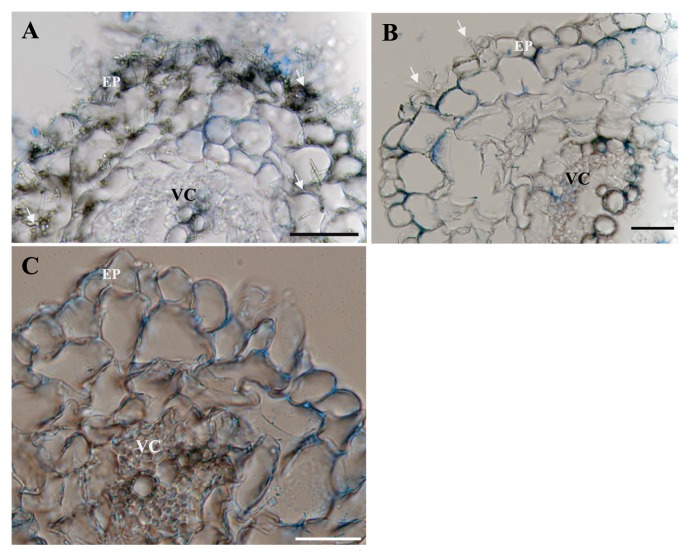
Colonization of Chinese cabbage roots by *Pseudosigmoidea ibarakiensis* sp. nov. isolate I.4-2-1. (A) Cross section of a Chinese cabbage root segment stained with 0.005% cotton blue in 50% acetic acid three weeks after inoculation showing heavy colonization in both epidermal and cortical layers. Arrows show fungal hyphae on the root surface, within epidermal cells (EP). VC=vascular cylinder. (B) Cross section of a Chinese cabbage root segment showing light colonization. (C) Cross section of an un-inoculated root segment serving as a control. Bars: 30 μm.

Cross sections of I.4-2-1-treated-Chinese cabbage roots showed after 20 days of incubation that the heavy infection of root tissues was limited to the epidermal and cortical layers of the hosts, but could not penetrate the vascular cylinder (Figs. 3A and 3B). These hyphae were mostly abundant in the epidermis and outer cortical layer although they could be found occasionally in the inner cortical layer (Fig. 3A). No visible signs of host reactions were seen in root cells colonized by the fungus. In the control, the epidermis and cortical cells remained intact during the observation period (Fig. 3C).

### Nitrogen source utilization and anatomic observations

**Fig. 4:**
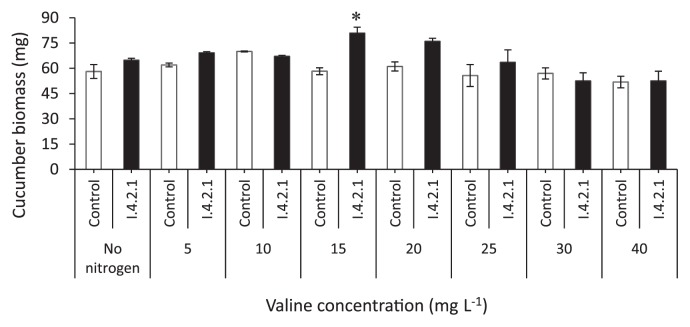
Dry weights of I.4-2-1-inoculated cucumber seedlings grown on oatmeal agar enriched with MgSO_4_·7H_2_O, KH_2_PO_4_, but no NaNO_3_, and amended with valine at various concentrations (Control: without endophytic fungus I.4-2-1). Data are the mean±SD, n=3. Asterisk indicates a significant difference from the control (P<0.05) following Tukey’s honestly significant difference test.

**Fig. 5:**
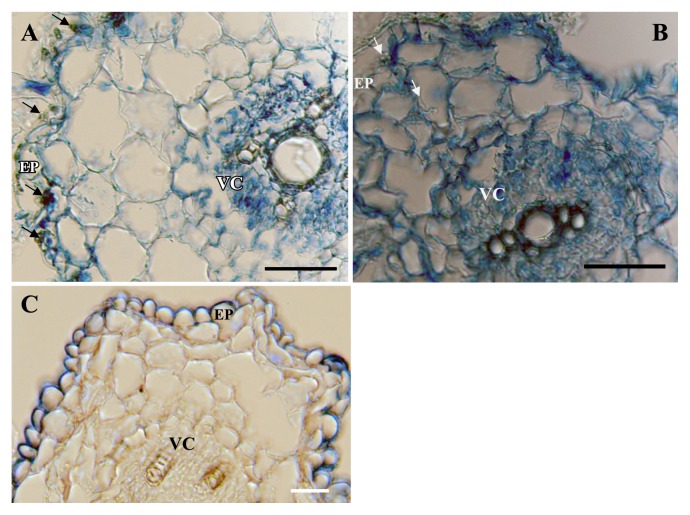
Colonization of cucumber roots by *Pseudosigmoidea ibarakiensis* sp. nov. isolate I.4-2-1 under conditions of substitution of NaNO3 by valine as a nitrogen source at various concentrations. (A) Cross section of a cucumber root segment stained with 0.005% cotton blue in 50% acetic acid three weeks after inoculation of 15 mg valine showing heavy colonization in epidermal cells. Arrows show fungal hyphae on the root surface, within epidermal cells (EP). VC=vascular cylinder. (B) Cross sections of a cucumber root segment showing light colonization after 40 mg valine. (C) Cross section of an uninoculated root segment serving as a control. Bars: 30 μm.

On OMA, isolate I.4-2-1 was able to grow successfully in all valine, phenylalanine and leucine treatments. On another hand, cucumber seedlings grown on these plates showed an increased plant biomass of 22% and 6% with valine and phenylalanine treatments, respectively, compared to the control (no nitrogen). As for leucine treatment, a 44% decrease of the cucumber seedling biomass was observed. Optimum plant growth was observed at 15 mg valine (Fig. 4). Anatomical observation showed that the fungus colonized heavily only in the epidermal and upper layer cells at the concentration of 15 mg valine (Fig. 5A), but slight colonization at 40 mg (Fig. 5B).

### pH tolerance tests and anatomic observations

**Fig. 6:**
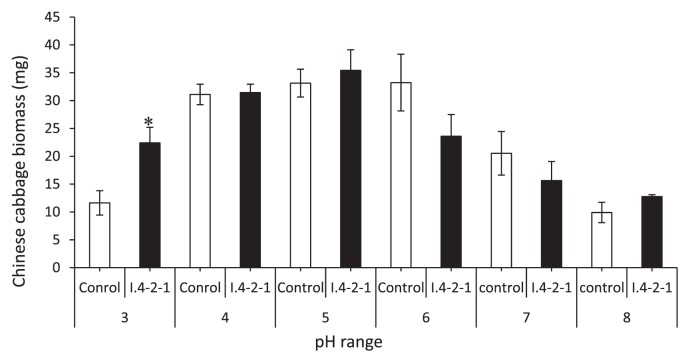
Effects of pH on the growth of Chinese cabbage plants inoculated with *Pseudosigmoidea ibarakiensis* sp.nov. and incubated at 23°C with a 18 h:6 h (L:D) photoperiod and 600 lux. Data are the mean±SD, n=3. Asterisk indicates a significant difference from the control (P<0.05) following Tukey’s honestly significant difference test.

Tests results showed that Chinese cabbage plants inoculated with I.4-2-1 grew well from pH 3 to 6 but not beyond. As for control plants, their growth was normal between pH 4 and 6. At pH 3, the inoculated plants grew significantly with a 92% increase in biomass over the control plants (Fig. 6). Anatomical observation showed that the fungus colonized heavily in the epidermal and upper layer cells under pH 3 (Fig. 3A), but slight colonization under pH 8 (Fig. 3B).

## Discussion

A new endophytic species of *Pseudosigmoidea* is described in this paper. This finding shows that other undescribed DSE species can be found in coniferous rainforests, characteristic of warmer temperate latitudes. The new species, *Pseudosigmoidea ibarakiensis*, showed endophytic features in the inoculation tests to some host plants, but its endophytic role in nature is still unknown. The habitat of this aquatic hyphomycete is reported to be submerged debris (2). Globally, aquatic hyphomycetes, despite being known to be ubiquitous in streams, are considered as the dominant mycobiota associated with decaying leaves (3, 11). Although some aquatic hyphomycetes, such as *Clavariopsis aquatic* and *Heliscus lugdunensis*, have been reported as root endophytes on living plants (32), they did not include any species of *Pseudosigmoidea* or *Sigmoidea*. Therefore, this study demonstrates for the first time the ability of a species of *Pseudosigmoidea* to grow as an endophyte on Chinese cabbage and other vegetables without causing any symptoms of disease or decay.

Several studies have proven the existence of fungal endophytes living in mutualistic relationships with host plants to which they provide essential nutrients that improve growth (9, 35, 36). The case of *Heteroconium chaetospira* transferring nitrogen to Chinese cabbage (35) can be taken as an example. However, there have been no reports of endophytes supporting plant growth for *Pseudosigmoidea*. Furthermore, this finding is important for the possibility of growing plants under conditions where nitrate (NaNO_3_) is replaced by l-phenylalanine or l-valine as a source of organic nitrogen. In addition, isolate I.4-2-1 conferred on Chinese cabbage plants the ability to grow at pH 3, which is classified as ultra-acidic (34), showing a 92% increase of biomass over control plants without DSE inoculation. In acidic soils, the problem of aluminum phytotoxicity is greatly increasing worldwide (41). Consequently, soil acidity is considered a serious growth-limiting factor to plants and can compromise crop production in up to 70% of the world’s potential arable land (13, 7). To improve these soils, liming is often used, but this is practically difficult and not sustainable. Although some mycorrhizae, such as *Pisolithus tinctorius* and other uncultivable endophytic fungi, are reported to play a role in the protection of plants in acidic and other contaminated soils (29, 41), there is no report of DSEs improving host plant growth under ultra-acidic conditions. Therefore, our finding is the first report of a DSE with the ability to support plant growth under ultra-acidic conditions. We did not extend our research to other DSE isolates to check whether they have the same ability to use organic nitrogen. One DSE, *H. chaetospira*, has already been reported to be able to transfer nitrogen to host plants (35). There are likely other DSEs able to use organic nitrogen sources or tolerate low pH as stress tolerance is reportedly a habitat specific phenomenon (31). Likewise, the mechanism by which this endophyte supports plant growth under low pH has not been addressed. However, this improvement of plant growth could be related to the ability of the isolate to use organic nitrogen sources under nitrogen-deficient conditions. Low nitrogen uptake is one of the effects associated with soil acidity (10, 26). The DSEs are thought to counteract the negative effects of high acidity on plants (28). The similarity of colonization patterns at low pH and optimum valine concentration suggests a relationship between the level of colonization and the impact on plant growth under unfavorable conditions.

Dark septate endophytes are known to colonize the intracellular and extracellular tissues of host plants tissues with no apparent negative effects (16). However, depending on the fungal species, the tissues colonized can differ and therefore have different impacts on the host plant. In contrast, the colonization of this species of *Pseudosigmoidea* is inter-and intracellular and limited to the epidermal and cortical layers, not reaching the vascular cylinder, causing no external symptoms or disruption of growth in host plants. No other test was undertaken to check the ability of environmental conditions to induce undesirable effects on host plants; however, we suggest that this isolate is sufficiently safe and the probability of undesirable effects on host plants remains very low.

Furthermore, the ability of this species of *Pseudosigmoidea* to colonize and grow in plants of four other families, Brassicae, Cucurbitacae, Solanaceae, and Poaceae, suggests a wide host range. DSEs, in general, have little or no host specificity. *Heteroconium chaetospira*, for example, is able to colonize the roots of plants from eight families (7, 25). Only barley, a gramineous crop, is not suitable for *H. chaetospira* (25). Although additional inoculation tests of this species of *Pseudosigmoidea* with different plant species under different environmental conditions are still needed before using this species, so far, there have been no negative reports advising against the use of this fungus in the commercial sector.

## Figures and Tables

**Table 1:** Numbers of slow-growing fungal isolates obtained from the bait samples of Chinese cabbage buried in cedar soils in Ibaraki

Isolation parameters	Collection sites with cedar trees and other dominant plant species				

	Site I	Site II	Site II	Site IV	Total
Total number of root segments	60	45	45	60	210
Total number of fungal isolates	23	14	26	30	93
Rate of fungal isolation (%)	38	31	58	50	44

NB: The rate of fungal isolation is calculated on the basis of the root segments analyzed as follows:
$$\frac{\text{Total number of fungal isolates}}{\text{Total number of root segments}}\times 100$$
